# Effect of Titanium Dioxide Particles on the Thermal Stability of Silica Aerogels

**DOI:** 10.3390/nano14151304

**Published:** 2024-08-02

**Authors:** Caide Fan, Jialu Lu, Chengjie Duan, Chengbin Wu, Jiming Lin, Ruoxiang Qiu, Zehui Zhang, Jianming Yang, Bin Zhou, Ai Du

**Affiliations:** 1 School of Physics Science and Engineering, Tongji University, Shanghai 200092, China; 2230948@tongji.edu.cn (C.F.); 21310191@tongji.edu.cn (J.L.); 2030956@tongji.edu.cn (C.W.); 1910105@tongji.edu.cn (Z.Z.); 1810908@tongji.edu.cn (J.Y.); 2 Shanghai Key Laboratory of Special Artificial Microstructure Materials and Technology, Tongji University, Shanghai 200092, China; 3 China Nuclear Power Technology Research Institute Co., Ltd., Shenzhen 518000, China; duanchengjie@cgnpc.com.cn (C.D.); qiuruoxiang@cngpc.com.cn (R.Q.); 4 Tsinghua Shenzhen International Graduate School, Tsinghua University, Shenzhen 518055, China

**Keywords:** silica aerogel, titanium dioxide particle, different crystal phase, high-temperature resistance, growth mode

## Abstract

Silica aerogels exhibit a unique nanostructure with low thermal conductivity and low density, making them attractive materials for thermal isolation under extreme conditions. The TiO_2_ particle is one of the common industrial additives used to reduce the thermal radiation of aerogel composites under high-temperature environments, but its influence on thermal resistance is almost unknown. Herein, we report the effect of TiO_2_ nanoparticles with different crystal phases and different sizes on the thermal stability of silica aerogel composites. By adding TiO_2_ nanoparticles, the aerogel can significantly resist collapse at high temperatures (up to 1000 °C). And compared with the rutile phase TiO_2_, the anatase phase TiO_2_ shows much higher temperature resistance performance, with shrinkage of only one-sixth of the rutile phase after 800 °C treatment. Interestingly, energy-dispersive spectrometer mapping results show that after 800 °C treatment, silica nanoparticles (NPs) are squeezed out in between anatase TiO_2_ particles, which resists the coarsening of silica NPs and ultimately enhances the stability of aerogel composites. The optimal anatase phase TiO_2_-doped silica aerogel demonstrates the integrated properties of crack-free morphology (2.84% shrinkage), low thermal conductivity (29.30 mW/(m·K)) and low density (149.4 mg/cm^3^) after 800 °C treatment. This study may provide new insights for developing oxide-doped silica aerogels with both high-temperature resistance and low thermal radiation.

## 1. Introduction

High-temperature protection is widely required in many fields, such as aerospace, nuclear power plants, new energy vehicles, factories, etc. [1,2,3,4] Compared to traditional materials such as rock wool and silica cryogel-glass fiber blankets [5,6,7], aerogels are favored for their low density and low thermal conductivity. Since the organosilicon source was introduced into the preparation of aerogels [8,9], the mechanical, thermal, optical, electrical, and acoustic properties of aerogels have been explored [10,11,12,13,14,15,16], among which the study of heat is the most fervent. Silica aerogel with super-thermal insulation can be synthesized by methltrimethoxysilane (MTMS), tetraethoxysilane (TEOS), tetramethoxysilane (TMOS), and some co-precursors [8,9,17,18,19]. Given the poor mechanical properties of TEOS- and TMOS-based aerogels, poly(methylsilsesquioxane) (PMSQ) aerogels based on methyl-containing precursors improve mechanical performance. However, methyl groups decompose at around 400 °C, leading to the destruction of the structure, which can seriously reduce thermal conductivity. And even for TEOS- and TMOS-based aerogels, the structure is coarsened at around 600 °C, significantly limiting the high-temperature applications of silica aerogels. [20]. In response, a series of metal oxide aerogels, such as alumina aerogels [21], zirconia aerogels [22], and alumina/zirconia oxide composite aerogels [23], have been explored to meet the high-temperature demand for aerogel. Nevertheless, the high cost and complexity of preparing metal oxide aerogels hinder their large-scale industrial dissemination. Thus, it appears more feasible to make some improvements to pure silica aerogels to make them suitable for higher-temperature environments.

Two main mechanisms constrain the promotion of silica aerogels for medium and high-temperature applications [24]. Firstly, the nanostructures of silica aerogel process high surface activity [20,25], making them prone to sintering above 650 °C. This sintering leads to nanoparticle growth and results in the volume shrinkage and cracking of aerogels, drastically limiting their utility at higher temperatures. Doping is an effective method to enhance the temperature resistance of aerogel skeletons. Therefore, the co-gelation method of metal oxides (such as zirconium oxide [26], alumina [27], yttrium oxide [28], etc.) and silica can be employed to improve the high-temperature resistance of silica oxide aerogels. However, these preparation processes tend to be complex, and the mechanical properties of as-prepared aerogels are often unsatisfactory, restricting their large-scale production and practical applications. Consequently, various high-temperature-resistant materials, such as silica nanowires [29], glass fibers [30], mullite nanosheet/titania nanorod [31], SiC fiber [32] and yttrium oxide nanoparticles [33], have been doped into silica aerogels. This approach not only improves the thermal properties of aerogel composites but also greatly enhances their mechanical properties. Secondly, thermal conductivity is influenced by solid conductivity, gas conductivity, and radiative conductivity [34,35,36]. Solid conductivity dominates at low temperatures, while at high temperatures, the contribution of heat radiation becomes more prominent. Most silica aerogels show a high transmission of infrared radiation. Thus, to decrease the thermal conductivity of silica aerogel at high temperatures, the use of an infrared opacifier becomes necessary. For instance, the chemical vapor infiltration(CVI) technique was applied by Daehee et al. [37] to add carbon to silica aerogels to improve their thermal performance by blocking infrared radiant heat transfer. Additionally, carbon nanotubes were used as an infrared opacifier by Jiang et al. [38] when doping Al_2_O_3_/SiO_2_ aerogels, resulting in a slight decrease in thermal conductivity at 1000 °C. However, both carbon and carbon nanotubes undergo oxidative decomposition at temperatures nearing 300 and 600 °C, respectively, which affects their performance at higher temperatures. In another study, Young-Geun et al. demonstrated that 5 wt% TiO_2_ powder-doped silica aerogel composites exhibited reduced thermal conductivity at 400 °C through opacification. [39] Similarly, Wang et al. [40] proposed a TiO_2_ powder-doped silica aerogel with a density of 0.26 g/cm^3^ and a thermal conductivity of 0.038 W/(m·K) at 800 K in air. It appears that titanium dioxide not only can enhance the mechanical properties of silicon oxide to minimize the high-temperature cracking of aerogels but also acts as an effective high-temperature infrared blocker.

Titanium dioxide has wide applications in various fields, such as dye adsorption [41], photocatalysis [42], sewage treatment, etc. The synthesis process for titanium dioxide nanoparticles is well-developed [43], enabling the production of particles with different sizes (from ten to a hundred nanometers) and various crystalline phases (rutile, anatase, and platelet titanium dioxide) as needed [44,45,46]. In the early 1990s, titanium dioxide particles were doped into aerogels to improve the thermal insulation effect of aerogels at high temperatures due to their good infrared barrier properties [47], which gradually became an industrial custom for infrared shielding. In most cases, TiO_2_ was used only for reducing thermal radiation under high-temperature environments. Although several studies in the literature point out the combination of silica aerogels and oxide additives could improve the high-temperature stability of the composites [26,27,28], fewer papers have studied the effect of oxides on the silica aerogel component and its corresponding mechanism. Moreover, the potential influence of infrared opacifiers like TiO_2_ on aerogel thermal resistance is rarely considered. Hence, investigating the doping of various titanium dioxide particles into aerogel for thermal resistance enhancement and the corresponding mechanism holds significant promise.

In this study, commercial titanium dioxide particles with different properties were successfully incorporated into silica aerogels, and their effects on the silica aerogels were investigated at both the macroscopic and microscopic levels. Additionally, possible explanations for observed discrepancies were proposed and verified. Finally, a corresponding theoretical model was established to explain these differences, providing valuable insights for aerogel composite design and high-temperature stability. This paper may provide a new idea for improving both the infrared shielding and thermal stability of aerogel composites by adding titania powder via a facile industrial process.

## 2. Materials and Methods

### 2.1. Materials and Reagent

The materials used mainly include tetraethylorthosilicate (TEOS), hydrofluoric acid (HF), absolute ethanol (EtOH), fumed silica and six kinds of TiO_2_ nanoparticles (60 nm rutile, 100 nm rutile, <100 nm rutile, 5–10 nm anatase, 100 nm anatase, <100 nm anatase) (Shanghai Aladdin Biochemical Technology Co., Ltd., AR, Shanghai, China). The deionized water was obtained from the College of Environmental Science and Engineering, Tongji University. All the materials were used as received without further purification.

### 2.2. Preparation of TiO_2_-Doped SiO_2_ Aerogel

The aerogels were prepared by a classic one-step and acid-base catalysis sol–gel process. TEOS was mixed with EtOH and deionized water at the volume ratio of 4:12:1; then, a certain amount of TiO_2_ nanoparticles and fumed silica were added into the solution and fully stirred for 30 min. The specific dosage and sample abbreviations are shown in Table 1. Take A-5–10 nm 0.6 as an example; here, A represents the crystalline phase of the particles (A for anatase and R for rutile), 5–10 nm represents the size of the doped particles and 0.6 represents the number of doped particles added. Then, we slowly dropped hydrofluoric acid as a catalyst into the mixture and poured it into a cylindrical mold after stirring. The gelation process was completed at room temperature for 10–20 min; then, an appropriate amount of ethanol was added to the surface to immerse the wet gel and sealed with a sealing film to prevent it from cracking due to solvent evaporation. The wet gel was placed in a thermostatic oven for 72 h at 40 °C to complete the aging. The resultant gel was replaced with ethanol at 40 °C three times for 8 h each time. After solvent replacement and surface modification, TiO_2_-doped SiO_2_ gels were supercritically dried in an autoclave (Shanghai Yanzheng GSH-10, Shanghai, China) at 10 L. Firstly, the wet gels with a volume of 0.35–0.4 L were immersed in 3 L of anhydrous ethanol; then, 2 L of high-purity nitrogen was flushed into the autoclave to generate an oxygen-free atmosphere as a safety precaution. Secondly, the autoclave was sealed tightly and heated from room temperature to 265 °C at a rate of 2 °C/min; the pressure of the autoclave rose to nearly 12 MPa, and the supercritical condition was kept for 1 h. Finally, supercritical ethanol was vented from the autoclave with a depressurization rate of 2 MPa/h, and the TiO_2_-doped SiO_2_ aerogels were obtained.

### 2.3. Thermal Treatment of TiO_2_-Doped SiO_2_ Aerogel

To determine the temperature resistance of different particle-doped aerogels, we placed the as-prepared aerogels in a muffle furnace at 400/600/800/1000 °C successively for heat treatment, maintaining each temperature for 4 h. After the heat treatment at each temperature, the samples were taken out for size and thermal conductivity measurements, and some samples were taken for microscopic morphology analysis.

### 2.4. Characterizations

The bulk density of the TiO_2_-doped SiO_2_ aerogels was determined by the weighting method. The line shrinkage of the material at 600/800/1000 °C was obtained by averaging the values with reference to the corresponding lengths of the material at room temperature. And the volume shrinkage of the material at 600/800/1000 °C was obtained with reference to the volume at room temperature. The morphology of the sample was characterized by transmission electron microscopy (TEM, Tecnai G2 F20 S-Twin, FEI Company, Hillsboro, OR, USA), and the elemental analysis was performed using energy-dispersive X-ray spectroscopy (EDS). The size distribution was obtained by counting the longer axes of the particles in the TEM using ImageJ software. Nitrogen adsorption–desorption isotherms at 77.4 K were measured using an N_2_ adsorption analyzer (TriStar 3000, Micromeritics, Norcross, GA, USA) to determine the specific surface area and pore size distribution. Before N_2_ adsorption/desorption measurements, the aerogel was degassed at 120 °C under a vacuum for approximately 12 h. The total specific surface area and the total pore volume were calculated using the BET equation applied at a relative pressure ranging from 0 to 1. The pore size distribution was obtained from the adsorption branch of the isotherms using the Barret–Joyner–Halenda (BJH) model. The phase analysis was measured by an X-ray diffractometer (XRD, Bruker D8-Discover, Bruker Company, Karlsruhe, Germany). Room temperature thermal conductivities were measured using a hotdisk thermal analyzer (TPS 3500, Hot Disk AB, Göteborg, Sweden). The thermal stability analysis was tested using a thermogravimetry analyzer coupled with a differential scanning calorimeter (TG-DSC, Q-600, TA instruments New Castle, DE, USA) at a heating rate of 10 °C/min under an airflow from 30 to 1000 °C.

## 3. Results and Discussion

### 3.1. Morphology Change in Samples under Different Temperature Treatment

**Figure 1:**
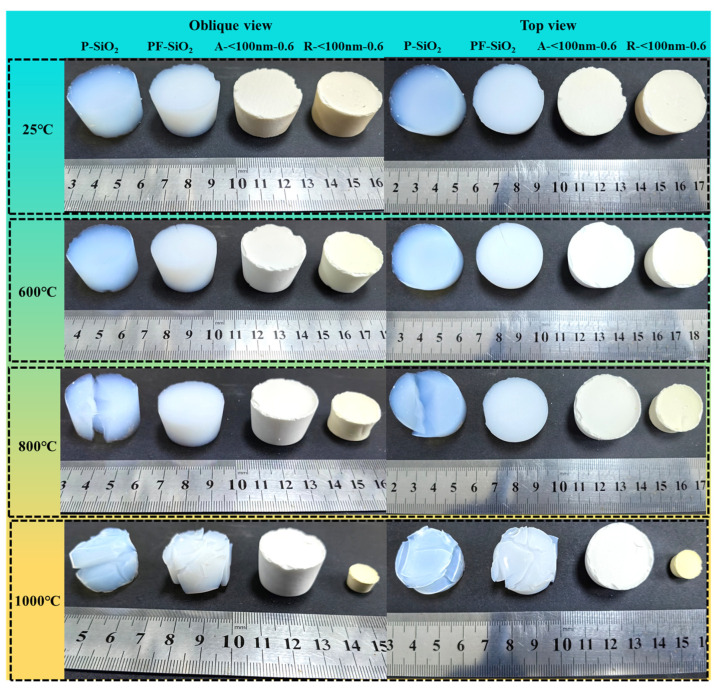
Oblique view and top view of morphology changes in samples treated at 25/600/800/1000 °C.

Herein, pure silica aerogel (P-SiO_2_), silica aerogel with the addition of fumed silica (PF-SiO_2_), and two types of aerogels doped with different titanium dioxide particles (R-<100 nm 0.6 for rutile phase titania and A-<100 nm 0.6 for anatase phase titania) was treated at 600/800/1000 °C for 4 h. The morphology of the samples after different temperature treatments is shown in Figure 1. It can be observed that the transparent aerogel turns white after the doping of the titanium dioxide nanoparticle, confirming that titanium dioxide is uniformly introduced into the silica skeleton successfully. This is further supported by the EDS and TEM results in Section 3.5 and Section 3.6. Taking the size and volume of each sample at room temperature as a reference, the corresponding line and volume shrinkage is demonstrated in Table 2. After 600 °C temperature treatment, only R-<100 nm 0.6 showed slight shrinkage (5.80% linear shrinkage rate), while the other samples showed no clear change in their apparent morphology. Following the treatment at 800 °C, P-SiO_2_ underwent obvious cracking, which surely led to a great increase in the thermal conductivity of the aerogel. The addition of fumed silica led to a certain enhancement of the mechanics of the aerogel, so the cracking could be avoided to maintain low gas conductivity and ensure the following characterizations, although it underwent a certain linear shrinkage of 5.12%. Meanwhile, the R-<100 nm 0.6 sample shrunk to 19.64% of its original diameter, but only 2.84%-dimensional change occurred for A-<100 nm 0.6. When the sintering temperature further increased, only the TiO_2_-doped samples were able to maintain their integrity. A-<100 nm 0.6 performs the best, and it seems that aerogels doped with anatase TiO_2_ have the best thermal stability. Anatase and rutile phase titania show dramatically different influences on the thermal stability of aerogel composites.

### 3.2. Effect of Particle Addition on Temperature Resistance of TiO_2_-Doped SiO_2_ Aerogels

**Figure 2:**
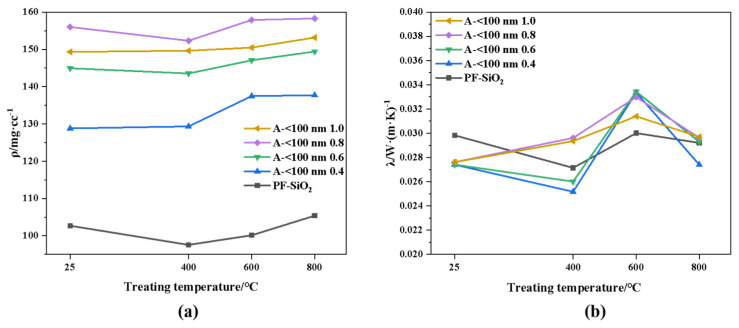
(**a**) Density and (**b**) thermal conductivity of aerogels with different particle additions treated at different temperatures.

Due to the outstanding temperature resistance of A-<100 nm 0.6, different amounts of anatase TiO_2_ particles with a size <100 nm were added to investigate the effect of particle addition on the high-temperature resistance performance of aerogels. They were treated at different temperatures for 4 h, and the corresponding thermal conductivity and density variation with temperature are shown in Figure 2. As shown in Figure 2a, the doping of titanium dioxide particles led to an increase in its density from 102.68 mg/cc to 149.34 mg/cc. It is worth noting that although the total mass increased, particle addition probably suppressed the shrinkage of the sample during supercritical drying. Thus, the density of the sample with 1.0 addition is slightly lower than that of 0.8. However, after different temperature treatments, the density only fluctuated around the initial density without significant changes, with its average fluctuation range at around 2.45%. Regarding thermal conductivity, as shown in Figure 2b, although the doping of the particles increased the solid heat transfer of the aerogel, the appropriate doping number of particles may lead to the good dispersity of silica aerogels, resulting in a slightly lower thermal conductivity (~2.54 mW/(m·K)) compared to the aerogels without particle doping. The thermal conductivity of the aerogel also fluctuates around the initial value after different temperature treatments, with the deviation relative to the aerogel without doping for no more than 4.38 mW/(m·K). It is not difficult to conclude that the different additions of particles only have a relatively large effect on the initial density of the aerogel but do not have much effect on the amount of density and thermal conductivity changes. Furthermore, although PF-SiO_2_ has a small density and thermal conductivity change after heat treatment, its thermal conductivity under high-temperature conditions is much larger than that of the aerogel without infrared blocker doping due to the large increase in radiative heat transfer, and that is the reason why the TiO_2_ particle was used.

### 3.3. Properties of Particles on the Thermal Stability of TiO_2_-Doped SiO_2_ Aerogels

**Figure 3:**
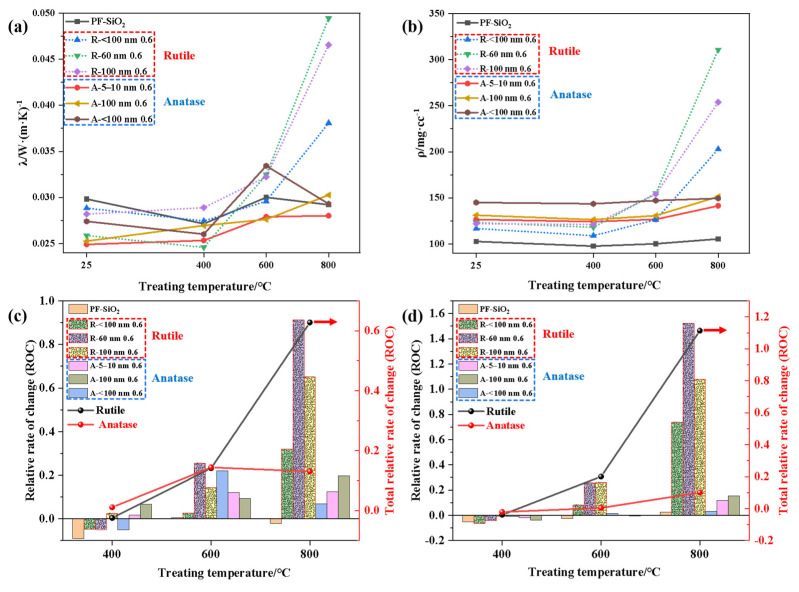
(**a**) Thermal conductivity and (**b**) density changes in samples treated at different temperatures. Relative rate of change in samples’ (**c**) thermal conductivity and (**d**) density treated at different temperatures.

Then, aerogels doped with TiO_2_ with different properties were treated at different temperatures for 4 h, and the density and thermal conductivity are displayed in Figure 3a,b. All the samples showed a slight decrease in density after treatment at 400 °C, which resulted from mass reduction due to the evaporation of water and decomposition of organics, which can be discussed in detail when analyzing TG-DSC results. The differences between rutile-TiO_2_- and anatase-TiO_2_-doped samples is amplified after 600 °C, where anatase-TiO_2_-doped aerogels show much better performance in terms of density and thermal conductivity. The rate of change (ROC) at different temperatures was obtained, and these are statistically shown in Figure 3c,d using the parameters at room temperature as a reference, i.e., *a* represents the thermal conductivity or density of the sample at 25 °C, *b* represents the sample’s thermal conductivity or density after treatment at 600 °C, then the ROC of thermal conductivity or density is expressed as (*b* − *a*)/*a* × 100%. For the aerogel without particle doping, its density and thermal conductivity do not change much in comparison with room temperature. For the particle doped with anatase TiO_2_, its average thermal conductivity changed little with temperature (less than 20%), while for the rutile particle-doped aerogels, its average thermal conductivity increased by 60% and density increased by 120%. It is evident that rutile-TiO_2_-doped aerogels undergo significant shrinkage after high-temperature treatment, resulting in a sharp increase in thermal conductivity. In contrast, anatase-TiO_2_-doped aerogels perform much better. It is clear that the effect of the particle size on the temperature resistance of the aerogels is far less significant than that of its crystalline phase on their heat resistance.

### 3.4. X-ray Diffraction Analysis of TiO_2_-Doped SiO_2_ Aerogels

**Figure 4:**
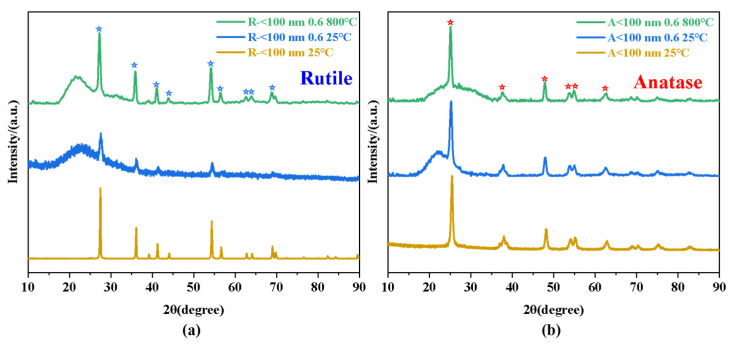
XRD results of the (**a**) R-<100 nm 0.6 and raw material (R-<100 nm 25 °C) and (**b**) A-<100 nm 0.6 and raw material (A-<100 nm 25 °C).

To further investigate the effect of titanium dioxide crystals on the temperature resistance of silica aerogels, two samples (R-<100 nm 0.6 and A-<100 nm 0.6) were chosen to study the different performance between anatase and rutile TiO_2_ particles on SiO_2_ aerogels. To ensure that the doping process did not lead to changes in the crystalline phase, XRD tests were first performed on the raw materials and as-prepared samples; the results are presented in Figure 4. The crystalline phases of raw materials are consistent with the product information, and no crystalline phase change occurred during the doping process. Compared to the raw materials, the aerogel doped with particles had a large peak at *θ* < 15°, which originated from the indeterminate peak from the silica, and thereafter, the peaks all originated from the titanium dioxide crystalline phase. Adding those two raw particles into the SiO_2_ aerogel, corresponding aerogels showed rutile and anatase phases, which proved that our doping process did not change the crystalline phase of titanium dioxide particles. Furthermore, the crystalline phases of the two samples after treatment at 800 °C were investigated, and we found that the crystalline phases of the two samples also remained unchanged. Despite the fact that anatase is an unstable phase, it did not undergo a phase transition within the scope of our study. It seems that phase transition is not responsible for the temperature resistance of aerogels, which might result from the relatively short treatment period and silicon’s ability to delay the anatase-rutile phase transition temperature [48,49,50,51]. So, it seems that phase transition is not responsible for the temperature resistance of aerogels.

### 3.5. Microscopic Morphology Analysis of TiO_2_-Doped SiO_2_ Aerogels

**Figure 5:**
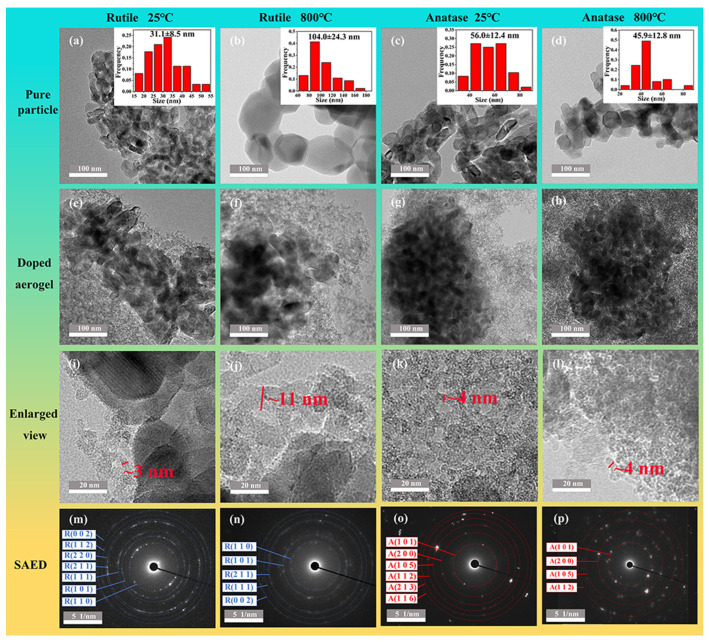
TEM of pure particles treated at different temperatures. (**a**) Rutile-<100 nm at 25 °C, (**b**) rutile-<100 nm at 800 °C, (**c**) anatase-<100 nm at 25 °C, and (**d**) anatase-<100 nm at 800 °C. Inserts show the corresponding particle size distribution. The TEM of doped aerogels treated at different temperatures is shown. (**e**) R-<100 nm 0.6 at 25 °C, (**f**) R-<100 nm 0.6 at 800 °C, (**g**) A-<100 nm 0.6 at 25 °C, and (**h**) A-<100 nm 0.6 at 800 °C. The high-resolution TEM of doped aerogels treated at different temperatures is shown. (**i**) R-<100 nm 0.6 at 25 °C, (**j**) R-<100 nm 0.6 at 800 °C, (**k**) A-<100 nm 0.6 at 25 °C, and (**l**) A-<100 nm 0.6 at 800 °C. The SAED of doped aerogels treated at different temperatures is shown. (**m**) R-<100 nm 0.6 at 25 °C, (**n**) R-<100 nm 0.6 at 800 °C, (**o**) A-<100 nm 0.6 at 25 °C, and (**p**) A-<100 nm 0.6 at 800 °C.

Figure 5a–d show the morphology and corresponding particle size distribution of the two raw materials after treatment at different temperatures, respectively. At room temperature, both particles are ellipsoidal, with R-<100 nm particles appearing slightly smaller than the A-<100 nm ones. After the treatment at 800 °C, the R-<100 nm particles grew from 31.1 nm to 104.0 nm, whereas the particles of R-<100 nm changed from ellipsoidal to spherical, and the average size of the particles even reduced from 56.4 nm to 45.6 nm as the results were obtained based on the longer axis. It can be seen that their temperature resistance is relatively better. The microscopic morphology and the corresponding high-resolution TEM of R-<100 nm 0.6 and A-<100 nm 0.6 samples treated at two different temperatures are shown in Figure 5e–l, where the slender silica skeleton grows around the TiO_2_ and the size of the TiO_2_ particles does not change significantly. The particles in the two samples grew to a certain extent after the treatment at 800 °C, but none of them changed as significantly as the pure particles, which originated from the protection of the particles by silica aerogels. Comparing the two samples with different particle doping, both TiO_2_ particles and the silicon dioxide skeleton of R-<100 nm 0.6 became larger after high-temperature treatment. Also, we observed that the interface between the particles and the silica skeleton became fuzzy, which originated from the softening of the silica and its impregnation into the clusters. The coarsening and softening of the skeleton together lead to an increase in aerogel density and thermal conductivity. However, for A-<100 nm 0.6, the TiO_2_ particles and silicon dioxide skeleton remained almost the same, resulting in its better performance in high temperature. Meanwhile, the selected area electron diffraction (SAED) pattern of R-<100 nm 0.6 and A-<100 nm 0.6 treated at two different temperatures are shown in Figure 5m–p and no partial phase transitions were detected, which is inconsistent with the XRD results.

### 3.6. Elemental Distributions Analysis of TiO_2_-Doped SiO_2_ Aerogels

**Figure 6:**
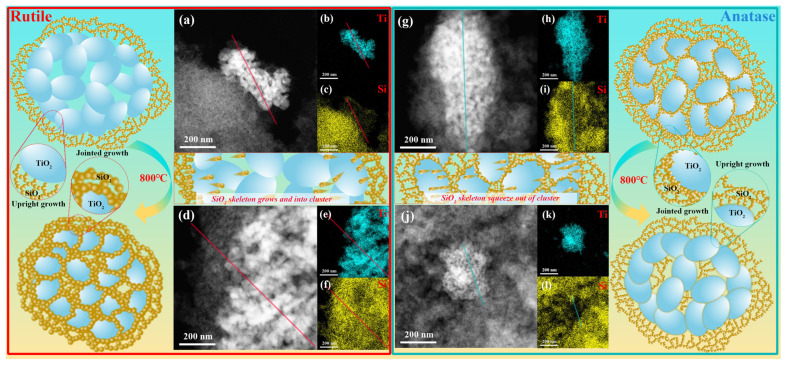
Elemental distribution analysis of doped TiO_2_ aerogels: the (**a**) image, (**b**) Ti and (**c**) Si distribution of R-<100 nm 0.6 at room temperature; the (**d**) image, (**e**) Ti and (**f**) Si distribution of R-<100 nm 0.6 after the treatment of 800 °C; the (**g**) image, (**h**) Ti and (**i**) Si distribution of A-<100 nm 0.6 at room temperature; the (**j**) image, (**k**) Ti and (**l**) Si distribution of A-<100 nm 0.6 after the treatment at 800 °C. Beside each figure is the corresponding schematic diagram; the blue ball represents TiO_2_ particles, and the yellow ball represents SiO_2_ particles. The lines in the figures correspond to the greyscale analysis of Figure 7.

**Figure 7:**
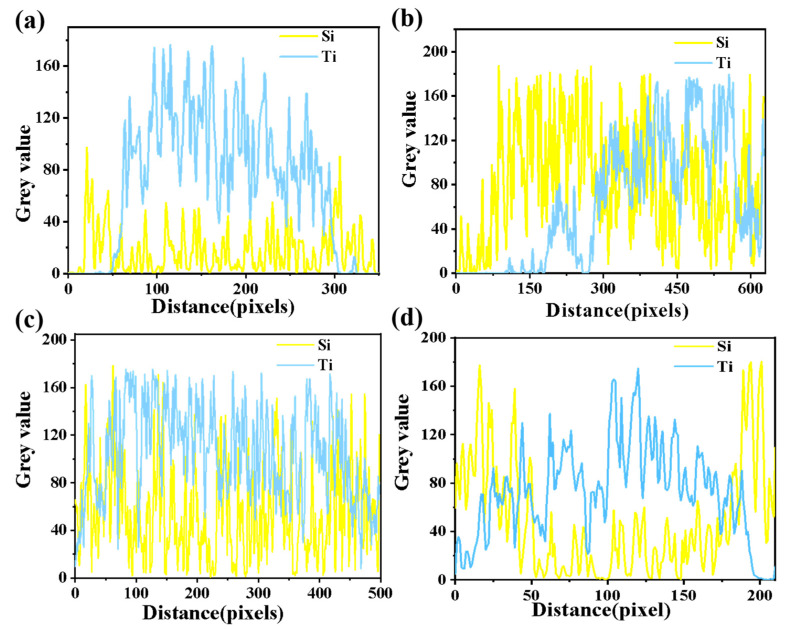
Distribution of Ti and Si along the straight line of Figure 6: (**a**) R-<100 nm 0.6 at 25 °C, (**b**) R-<100 nm 0.6 at 800 °C, (**c**) A-<100 nm 0.6 at 25 °C, and (**d**) A-<100 nm 0.6 at 800 °C.

The corresponding elemental distributions of the samples after different temperature treatments are illustrated in Figure 6. The distribution of silicon in the particle clusters is significantly different, with the rutile-phase particle clusters containing less silicon in and around them, while anatase-phase particle clusters contained more silicon. After heat treatment, the rutile-phase particle clusters were filled with silicon in their interiors, while the anatase-phase particle clusters contained even less silicon than before. To further visualize this process, a straight line through the clusters of titanium dioxide particles was drawn and is shown in Figure 6. Then, the grey values of Si and Ti along them were obtained using ImageJ software, and the results are shown in Figure 7. Two different distribution patterns can be clearly seen: the distributions of silicon and titanium are irregular, and the grey value is comparable when the infiltration is better (R-<100 nm 0.6 at 800 °C and A-<100 nm 0.6 at 25 °C); conversely, when the infiltration is poor (R-<100 nm 0.6 at 25 °C and A-<100 nm 0.6 at 800 °C), silicon’s grey value at the position corresponding to titanium is lower than titanium’s. The heat treatment process draws the silicon distributed on the surface of rutile clusters into the interior and expels the silicon uniformly distributed within anatase clusters to the surface; similar changes in Si distribution in anatase-TiO_2_ have been described by Okada et al. [51].

Inspired by Zhang [52], we hypothesize that the different growth modes and the affinity of particles for silicon at high temperatures can lead to the different performances of these two aerogels doped with rutile and anatase TiO_2_ particles. For TiO_2_ particles in the rutile phase, TiO_2_ particles are exclusive to silica at room temperature, and silicon dioxide tends to grow perpendicular to the surface of TiO_2_ particles, creating a certain air gap between the particles and the silica. Heat acts directly on the TiO_2_ particles through convection, leading to particle growth and the softening of the silica skeleton. The particles exhibit a better affinity for silicon at high temperatures, drawing softening silicon into the interiors. This considerably disrupts the microstructure of the aerogel, leading to a sharp increase in thermal conductivity and density. For anatase TiO_2_ particles, the infiltration of silica with TiO_2_ is much better at room temperature. Silicon dioxide adheres and grows on the surface of the particles, forming a protective layer to mitigate the impact of heat on titanium dioxide particles, thus slowing down heat conduction directly. The microstructure is preserved; hence, the thermal conductivity and density also remain almost unchanged. Additionally, the particles have less affinity for silicon at high temperatures, draining silicon towards the outside of the cluster and reducing the internal silicon content.

### 3.7. BET Analysis of TiO_2_-Doped SiO_2_ Aerogels

**Figure 8:**
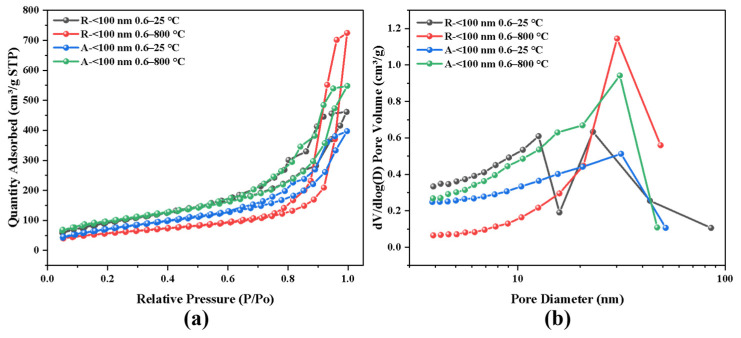
BET results of TiO_2_-doped aerogels: (**a**) Nitrogen adsorption–desorption isotherms of R-<100 nm 0.6 and A-<100 nm 0.6; (**b**) Pore size distribution of R-<100 nm 0.6 and A-<100 nm 0.6.

To further support our conjecture, BET tests were subjected to these two samples treated with different temperatures, and the results are displayed in Figure 8. As seen from the adsorption and desorption curves shown in Figure 8a, according to the classification of IUPAC, the adsorption isotherms of all samples before and after heat treatment are type IV curves, indicating that all the samples are mesoporous materials. The specific surface areas and average pore sizes are listed in Table 3. At room temperature, R-<100 nm 0.6 has a higher specific surface area than A-<100 nm 0.6 due to the silica aerogel skeleton growing upright on the surface of rutile particles while adhering to the surface of anatase. After heat treatment at 800 °C, the rutile particles inhaled the molten silica into the interior of the particle clusters, and the skeleton was greatly thickened, leading to a significant decrease in the specific surface area. In contrast, the particles of anatase unexpectedly showed an increase in the specific surface area due to their low affinity for silica at high temperatures. This led to the exclusion of silica from the particles of more pore structures, while the silica skeleton remained almost unchanged. For the pore size distribution shown in Figure 8b, the samples without high-temperature treatment generally have wider pore structures, while the pore distribution becomes more concentrated after heat treatment. Due to the poor temperature resistance of R-<100 nm 0.6, its skeleton clearly grows, resulting in a significant increase in pore size. The less skeleton growth that occurred in A-<100 nm 0.6 and the exclusion of silicon from the particle clusters resulted in a pore structure, leading to an almost unchanged average pore size.

### 3.8. TG-DSC Analysis of SiO_2_- and TiO_2_-Doped SiO_2_ Aerogels

**Figure 9:**
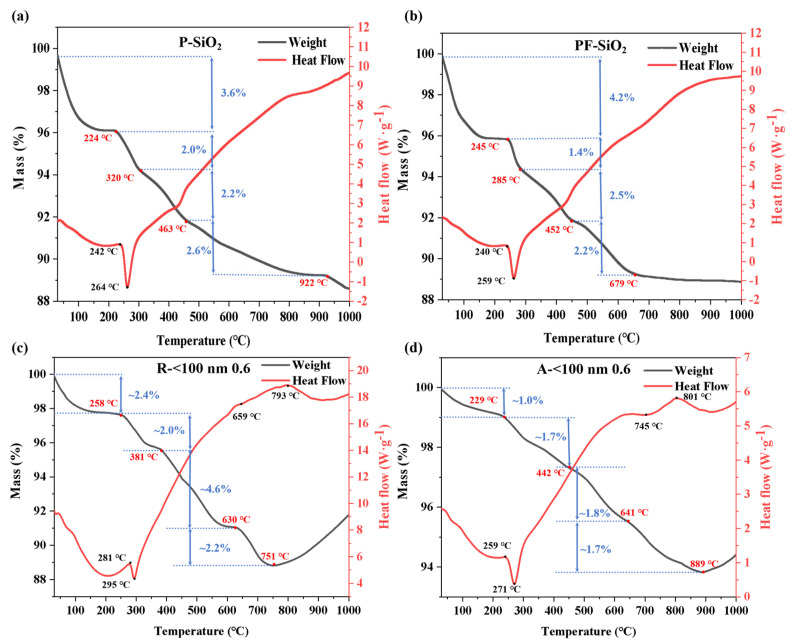
TG-DSC results of (**a**) P-SiO_2_, (**b**) PF-SiO_2_, (**c**) R-<100 nm 0.6, and (**d**) A-<100 nm 0.6.

In order to understand the dynamics of the heat treatment process, we show the obtained TG-DSC results in Figure 9 and divide the heat treatment process into five parts. For P-SiO_2_, weight loss processes probably arose from the evaporation of absorbed water or other volatile matters (about 30–220 °C), the decomposition of residual organic matter (about 220–320 °C, with an endothermic peak at ~260 °C), dehydration of OH groups and decomposition of methyl groups produced by the supercritical process (about 320–460 °C), the phase transition of SiO_2_ and possible surface reactions (about 460–920 °C), and further connections between chemical bonds (about 920–1000 °C) [41,53]. For PF-SiO_2_, the addition of fumed silica caused an advance in the last stage. However, as a common additive of aerogel composites in the industry, it was effective at improving the mechanics of the pure silica aerogel to prevent heating cracking, and its thermal conductivity was held at a low level. For the R-<100 nm 0.6 and A-<100 nm 0.6 samples, the addition of titanium dioxide slowed down the first three stages. In addition, the crystallization and oxidation of TiO_2_ (last stage) lead to an increase in mass. The sample A-<100 nm 0.6 shows only half the mass loss of rutile one for 400–650 °C, probably due to the better bonding of anatase-phase titanium dioxide to silicon oxide, leading to fewer functional groups on the silicon surface. Moreover, during 650–800 °C, the heat flow difference may be derived from the association (rutile, R-<100 nm 0.6) or segregation (anatase, A-<100 nm 0.6) of SiO_2_ and TiO_2_, which is in accordance with SEM and EDS mapping results.

Overall, both samples have more thermal weight loss as a whole, indicating their good thermal stability. However, it can be clearly seen that the sample doped with titanium dioxide particles in the anatase phase is superior to that doped with rutile one, which is mainly indicated by its lower mass loss, higher glass transition temperature, and fewer changes in heat flow.

## 4. Conclusions

In this work, different titanium dioxide particles were successfully and uniformly doped into silica aerogels. Unlike many studies that have focused on the properties of titanium dioxide particles, we investigated the effect of titanium dioxide particle doping on the temperature resistance and mechanical properties of the aerogel and proposed certain conjectures to explain our observations. From our findings, we can draw the following conclusions:

- As one of the most commonly used infrared shading agents in silicon oxide aerogels, commercial titanium dioxide particles can provide stress points for the aerogel skeleton and significantly improve the mechanical properties. Consequently, the silica aerogels showed no crack under high-temperature treatment.
- The size and addition of titanium dioxide particles had a minimal effect on the temperature resistance of the aerogel. However, the crystal phase of the particles had a significant impact on the temperature resistance of the aerogel. The thermal conductivity and density variation results showed that the anatase titanium dioxide particles performed better than the rutile ones. We attribute this discrepancy to the different modes in which silicon oxide grows on the particle surface. Initially, the silicon oxide grows upright on the surface of rutile particles and adheres to the surface of anatase particles. However, under high-temperature conditions, the rutile draws molten silicon oxide into the particle clusters, leading to structural collapse, while the anatase expels the silicon oxide. This phenomenon is further confirmed by the XRD, EDS, TEM, and TG-DSC results.

These findings offer valuable insights for researchers selecting titanium dioxide infrared shading agents and pave the way for future advancements in aerogel technology.

## Figures and Tables

**Table 1:** Specific dosage for the preparation and sample abbreviations of TiO_2_-doped SiO_2_ aerogels.

Sample	TEOS (mL)	EtOH (mL)	H_2_O (mL)	HF (mL)	Fumed Silica (g)	TiO_2_ (g)	Particle Properties
P-SiO_2_	15	30	3.75	0.6	0	0	None
PF-SiO_2_	15	30	3.75	0.6	0.1	0	None
R-<100 nm 0.6	15	30	3.75	0.6	0.1	0.6	<100 nm Rutile
R-60 nm 0.6	15	30	3.75	0.6	0.1	0.6	60 nm Rutile
R-100 nm 0.6	15	30	3.75	0.6	0.1	0.6	100 nm Rutile
A-5–10 nm 0.6	15	30	3.75	0.6	0.1	0.6	5–10 nm Anatase
A-100 nm 0.6	15	30	3.75	0.6	0.1	0.6	100 nm Anatase
A-<100 nm 0.4	15	30	3.75	0.6	0.1	0.4	<100 nm Anatase
A-<100 nm 0.6	15	30	3.75	0.6	0.1	0.6	<100 nm Anatase
A-<100 nm 0.8	15	30	3.75	0.6	0.1	0.8	<100 nm Anatase
A-<100 nm 1.0	15	30	3.75	0.6	0.1	1.0	<100 nm Anatase

**Table 2:** Size shrinkage and volume shrinkage of P-SiO_2_, PF-SiO_2_, A-<100 nm 0.6, and R-<100 nm 0.6 after different temperature treatments.

		600 °C	800 °C	1000 °C
P-SiO_2_	linear shrinkage	1.20%	None *	None
	volume shrinkage	5.21%	None	None
PF-SiO_2_	linear shrinkage	2.10%	5.12%	None
	volume shrinkage	6.18%	14.54%	None
A-<100 nm 0.6	linear shrinkage	1.30%	2.84%	26.92%
	volume shrinkage	0.28%	3.81%	61.57%
R-<100 nm 0.6	linear shrinkage	5.80%	19.64%	62.50%
	volume shrinkage	17.06%	49.03%	94.76%

* None denotes the cracking of the sample volume after treatment at the corresponding temperature.

**Table 3:** Pore structure information of R-<100 nm 0.6 and A-<100 nm 0.6 at different temperatures.

Sample	Surface Area of Pores (m^2^/g)	Total Pore Volume (cm^3^/g)	Average Pore Diameter (nm)
R-<100 nm 0.6–25 °C	342.2193	0.612398	12.9404
R-<100 nm 0.6–800 °C	207.6177	1.086012	29.7422
A-<100 nm 0.6–25 °C	273.6364	0.532913	14.4016
A-<100 nm 0.6–800 °C	354.3172	0.752726	14.9889

## Data Availability

The data presented in this study are available on request from the corresponding authors.
